# Voice analysis as an objective state marker in bipolar disorder

**DOI:** 10.1038/tp.2016.123

**Published:** 2016-07-19

**Authors:** M Faurholt-Jepsen, J Busk, M Frost, M Vinberg, E M Christensen, O Winther, J E Bardram, L V Kessing

**Affiliations:** 1 Psychiatric Center Copenhagen, Rigshospitalet, Copenhagen, Denmark; 2 DTU Compute, Technical University of Denmark (DTU), Lyngby, Denmark; 3 The Pervasive Interaction Laboratory, IT University of Copenhagen, Copenhagen, Denmark

## Abstract

Changes in speech have been suggested as sensitive and valid measures of depression and mania in bipolar disorder. The present study aimed at investigating (1) voice features collected during phone calls as objective markers of affective states in bipolar disorder and (2) if combining voice features with automatically generated objective smartphone data on behavioral activities (for example, number of text messages and phone calls per day) and electronic self-monitored data (mood) on illness activity would increase the accuracy as a marker of affective states. Using smartphones, voice features, automatically generated objective smartphone data on behavioral activities and electronic self-monitored data were collected from 28 outpatients with bipolar disorder in naturalistic settings on a daily basis during a period of 12 weeks. Depressive and manic symptoms were assessed using the Hamilton Depression Rating Scale 17-item and the Young Mania Rating Scale, respectively, by a researcher blinded to smartphone data. Data were analyzed using random forest algorithms. Affective states were classified using voice features extracted during everyday life phone calls. Voice features were found to be more accurate, sensitive and specific in the classification of manic or mixed states with an area under the curve (AUC)=0.89 compared with an AUC=0.78 for the classification of depressive states. Combining voice features with automatically generated objective smartphone data on behavioral activities and electronic self-monitored data increased the accuracy, sensitivity and specificity of classification of affective states slightly. Voice features collected in naturalistic settings using smartphones may be used as objective state markers in patients with bipolar disorder.

## Introduction

Observer-based clinical rating scales such as the Hamilton Depression Rating Scale 17-item (HAMD)^1^ and the Young Mania Rating Scale (YMRS)^2^ are used as golden standards to assess the severity of depressive and manic symptoms when treating patients with bipolar disorder. However, using these clinical rating scales requires clinician–patient encounter. Further, the severity of depressive and manic symptoms is determined by a subjective clinical evaluation in a semi-structured interview with the risk of individual observer bias. Developing objective and continuous measures of symptoms’ severity to assist the clinical assessment would be a major breakthrough.^3^, ^4^ Methods using continuous and real-time monitoring of objectively observable data on illness activity in bipolar disorder that would be able to discriminate between affective states could help clinicians to improve the diagnosis of affective states, provide options for early intervention on prodromal symptoms, and allow for close and continuous monitoring and collection of real-time data on depressive and manic symptoms outside clinical settings between outpatient visits.

Studies analyzing the spoken language in affective disorders date back as early as 1938.^5^ A number of clinical observations suggest that reduced speech activity and changes in voice features such as pitch may be sensitive and valid measures of prodromal symptoms of depression and effect of treatment.^6^, ^7^, ^8^, ^9^, ^10^, ^11^, ^12^ Conversely, it has been suggested that increased speech activity may predict a switch to hypomania.^13^ Item number eight on the HAMD (psychomotor retardation) and item number six on the YMRS (speech amount and rate) are both related to changes in speech, illustrating that factors related to speech activity are important aspects to evaluate in the assessment of symptoms’ severity in bipolar disorder. Based on these clinical observations there is an increasing interest in electronic systems for speech emotion recognition that can be used to extract useful semantics from speech and thereby provide information on the emotional state of the speaker (for example, information on pitch of the voice).^14^

Software for ecologically extracting data on multiple voice features during phone calls made in naturalistic settings over prolonged time-periods has been developed^15^ and a few preliminary studies have been published.^16^, ^17^, ^18^, ^19^, ^20^ One study extracted voice features in six patients with bipolar disorder type I using software on smartphones and demonstrated that changes in speech data were able to detect the presence of depressive and hypomanic symptoms assessed with weekly phone-based clinicians administrated ratings using the HAMD and the YMRS, respectively.^17^ However, none of the patients in the study presented with manic symptoms during the study period, and the clinical assessments were phone-based. Another study on six patients with bipolar disorder showed that combining statistics on objectively collected duration of phone calls per day and extracted voice features on variance of pitch increased the accuracy of classification of affective states compared with solely using variance of pitch for classification.^18^, ^19^ The study did not state if and how the affective states were assessed during the monitoring period.

In addition to voice features, changes in behavioral activities such as physical activity/psychomotor activity^21^, ^22^, ^23^, ^24^ and the level of engagement in social activities^25^ represent central aspects of illness activity in bipolar disorder and these can be objectively evaluated using smartphones as demonstrated by our group.^26^, ^27^, ^28^

In 2010 an electronic monitoring system for smartphones (the MONARCA system) for patients with bipolar disorder was developed by the authors.^29^, ^30^, ^31^ The system allows for daily electronic self-monitoring of subjective items reflecting illness activity (for example, mood, sleep length, activity level, medicine intake) and collection of automatically generated objective data on different aspects of behavioral activities (for example, the number and duration of incoming and outgoing of phone calls; the number of incoming and outgoing text messages (social activities); accelerometer data (physical activity); the amount of movement between cell tower IDs (mobility); and the number of times and duration the smartphone’s screen is turned ‘on’ (phone usage). Studies on patients with bipolar disorder using the MONARCA system showed that automatically generated objective data collected using smartphones correlate with the severity of clinically rated depressive and manic symptoms. Further, the studies showed that automatically generated objective data discriminate between affective states, and daily electronic self-monitored items reflecting illness activity (for example, self-monitored mood) correlate with the severity of clinically rated depressive and manic symptoms.^26^, ^27^, ^28^

Recently, the MONARCA system was extended to collect and extract voice features from phone calls made during everyday life in naturalistic settings.

Using this new version of the MONARCA system in patients with bipolar disorder presenting with moderate to severe levels of depressive and manic symptoms, the objectives of the present longitudinal study were to test the following hypotheses: (1) voice features extracted during phone calls from everyday life in naturalistic settings would be able to discriminate between affective states, and (2) combining voice features with automatically generated objective data on different aspects of behavioral activities and electronic self-monitored data would increase the accuracy of discriminating between affective states.

## Materials and methods

### Study participants and settings

The patients were recruited from The Copenhagen Clinic for Affective Disorders, Psychiatric Center Copenhagen, Denmark,^32^ during the period of October 2013 to December 2014.

Inclusion criteria were: bipolar disorder diagnosis according to ICD-10 using the Schedules for Clinical Assessment in Neuropsychiatry (SCAN) interview.^33^ Exclusion criteria were: pregnancy; lack of Danish language skills; and schizophrenia, schizotypal or delusional disorders according to the SCAN interview. The patients participated in the study for a period of 12 weeks during the early phase of their course of treatment at the clinic and received various types, doses and combinations of psychopharmacological treatment. Patients were invited to participate in the study following referral to the clinic and clinical and socio-demographic data were collected at inclusion.

The patients either used their own Android smartphone or were offered to loan an Android smartphone (HTC Desire S, New Taipei City, Taiwan or LG Nexus 5, Seoul, South Korea), free of charge during the study period. The patients used their own SIM card and did not receive economic compensation for participating in the study. The patients were instructed to use the smartphone for their usual communicative purposes, to use the smartphone as their primary phone and to carry it with them during the day as much as possible.

### Electronic self-monitored data

The self-monitoring part of the MONARCA app was installed on the smartphones and made an alarm sound once a day, at a time chosen by the patients, to prompt the patients to provide electronic self-monitored data. If the patient forgot to provide data it was possible to do so retrospectively for up to 2 days. Retrospectively collected data were marked as such in the MONARCA system.

The following self-monitored parameters were evaluated on a daily basis by the patients: mood (scored from depressive to manic on a scale from −3 to +3, including scores of +0.5 and −0.5); sleep length (number of hours slept/night measured in half hours intervals); medication taken (yes/no); medication taken with changes (yes/no); activity level (scored on a scale from −3 to +3); alcohol consumption (number of units per day); mixed mood (yes/no); irritability (yes/no); cognitive problems (yes/no); stress level (scored on a scale from 0–2); and indication of the presence of individualized early warning signs (yes/no).

### Automatically generated objective data

On a daily basis, automatically generated objective data on different aspects of behavioral activities were collected throughout the study period. The data collection did not require the patients to actively interact with the MONARCA software in any way. The level of social activity was reflected by data on the number of incoming and outgoing text messages; the duration of phone calls; and the number of incoming and outgoing phone calls. The level of mobility was reflected by data on the number of changes in cell tower IDs (reflecting movement between cell tower IDs) and the number of unique cell tower IDs. Data on the number of times and duration the smartphones’ screens were turned ‘on’ reflected the level of phone usage.

### Voice features

Voice features were extracted from the patients’ phone calls during everyday life in naturalistic settings using the open-source Media Interpretation by Large feature-space Extraction (openSMILE) toolkit,^15^ which is a feature extractor for signal processing and machine learning applications. It is designed for real-time online processing, but can also be used offline. In the present study, the toolkit ran directly on the patients’ smartphones, and the extracted features were encrypted, transmitted to a secure server and stored in a database for later data analyses. To collect as many features as possible the openSMILE toolkit was configured to use The large openSMILE emotion feature set (emolarge), which has a standard configuration of 6552 numerical features reflecting data on pitch, variance and so on. All the features in this configuration are not documented in the toolkit, but includes a large number of derived features such as mean, range, s.d., quartiles, inter-quartile range, descriptors and their delta regression coefficients.^15^

### Clinical assessments

The bipolar disorder diagnosis according to ICD-10 was confirmed using SCAN.^33^ Patients were invited to visit the researcher (MFJ) fortnightly during the 12-week study period. Affective states were defined according to an ICD-10 diagnosis of bipolar disorder current episode depressive, manic or mixed in combination with a total score of depressive and manic symptoms⩾13 according to standardized semi-structured interviews using the rating scales HAMD ^1^ and the YMRS,^2^ respectively. The cut-off on the HAMD and YMRS of 13 in contrast to a lower cut-off was chosen *a priori* to increase the validity of a current affective depressive or manic/mixed state (the more severe, the higher the validity). A current euthymic state was defined as a HAMD and YMRS <13 and in this way also including affective states with partial remission. The researcher (MFJ) did not have access to the automatically generated objective data and the extracted voice features collected by the smartphones during the study period, and thus was blinded to all objective smartphone data.

### Statistical methods

Clinical rating with the HAMD and the YMRS included the days of the rating and the 3 previous days. Consequently, we analyzed data on voice features, automatically generated objective data and electronic self-monitored data for the day of the clinical assessments of depressive and manic symptoms using the HAMD and the YMRS, respectively, and the 3 previous days. The patients’ affective states were categorized according to scores on the clinical rating scales into a euthymic state (HAMD<13 and YMRS<13); a depressive state (HAMD⩾13 and YMRS<13); and a manic or mixed state (YMRS⩾13).

Machine learning techniques aim to minimize error on held-out data by making a compromise between minimizing error on training set and penalizing model complexity. If the classes are very imbalanced (for example, few depression or mania versus euthymia) the solution found may become trivial (for example, only classifying euthymia).

In many cases, we observed class imbalance; one class was represented by a large amount of examples, while the other was represented by a few examples. To mitigate this problem, random oversampling, sampling the minority class with replacement, was used to create a balanced training set before learning the classifier. The Random Forest classifier combines several decision tree classifiers into a single classifier (the ‘forest’). Each tree is generated from a subsample of the training data and using a random subset of features to ensure maximal degree of independence among the trees. The combined classification is performed by majority voting, lowering the overall variance of the classifier thus preventing overfitting. The Random Forest classification algorithm was chosen because it tends to be good at handling datasets with many features, as tree induction methods automatically choose the most discriminating features in the data.^34^

Model evaluation was done by observing the performance of a classifier when applied to a set of previously unseen examples specifically reserved for testing (a test set). To assess the performance of a classifier, the accuracy/the percentage of examples that were classified correctly was calculated and defined as accuracy=(true positive+true negative)/(positive+negative). The sensitivity was calculated as true positive/positive, and the specificity was calculated as true negative/negative. In receiver operating characteristic curves (ROC), we assessed the performance of a binary classifier (depression versus euthymia; mania versus euthymia according to a cut-off on the HAMD and YMRS of ⩾13, respectively), and visualized the trade-off between the true positive rate (TPR/sensitivity) on the *y*-axis and the false-positive rate (FPR) (1- specificity) on the *x*-axis. The vertical axis of the ROC curve represents the TPR while the horizontal axis represents the FPR. Area under the curve (AUC) was used as a metric to assess the performance of a model.

*K*-fold cross-validation is a technique for estimating the performance based on randomly sampled partitions of the data. Data were randomly partitioned into *k* mutually exclusive subsets of approximately equal size. Training and testing was then performed k times, where in each iteration one partition was reserved as the test set and the remaining *k*−1 partitions form the training set. The overall accuracy estimate was computed as the average of the accuracies for each fold. Analysis was performed using both a user-dependent model, that is, building a model for each individual patient, and a user-independent model, that is, building a common model from all patients.

We evaluated the ability to classify affective states building four different models including (1) voice features exclusively, (2) voice features combined with automatically generated objective data, (3) voice features combined with daily electronic self-monitoring data, and (4) voice features combined with automatically generated objective data and daily electronic self-monitoring data.

Data on clinical assessments and socio-demographic data were entered using the data entry program Epidata (The EpiData Association, Odense, Denmark), and the computer language Python with the scikit-learn library and STATA version 12.1 (StataCorp, College Station, TX, USA) were used for data processing and analyses.

### Ethical considerations

The study was approved by the Regional Ethics Committee in the Capital Region of Denmark (H-2-2011-056) and the Danish Data protection agency (2013-41-1710). All potential participants were given both written and oral information about the study before informed consent was obtained.

## Results

### Background characteristics

During the period from October 2013 to December 2014, 51 eligible patients with a diagnosis of bipolar disorder according to ICD-10 were invited to participate in the present study; and of these, 32 (62.7%) were willing to participate in the study. The main reasons for declining to participate were that (1) it would be time-consuming (*N*=13) and 2) that the monitoring system was not available for iPhones (*N*=5). One patient declined to participate due to the collection of voice features and automatically generated objective data. Three patients dropped out of the study immediately after inclusion (changed their minds regarding participation in a scientific study). Consequently, 29 patients participated, but one patient did not provide data on voice features leaving a total of 28 patients available for the statistical analyses. A total of 8.7% (17 out of 196) of the patients’ visits with the researcher for assessment of the severity of depressive and manic symptoms using the HAMD and the YMRS, respectively, were missing, leaving 179 clinical ratings of depressive and manic symptoms available for the analyses.

The patients had a mean age of 30.3 (s.d. 9.3) years, a mean illness duration of 9.6 (s.d. 6.3) years and 65% (*N*=18) were women. Further information on the clinical and socio-demographic characteristics of patients are presented in Table 1. Table 2 presents the severity of depressive and manic symptoms according to affective states (depressive state, manic or mixed state and euthymic state) during the 12-week study period as represented by raw and unadjusted mean scores and s.d. of the HAMD and the YMRS, respectively. Of the 28 patients, 13 patients provided enough voice feature data to train at least one model for classification of affective states.

### Voice features for classification of affective states

**Figure 1:**
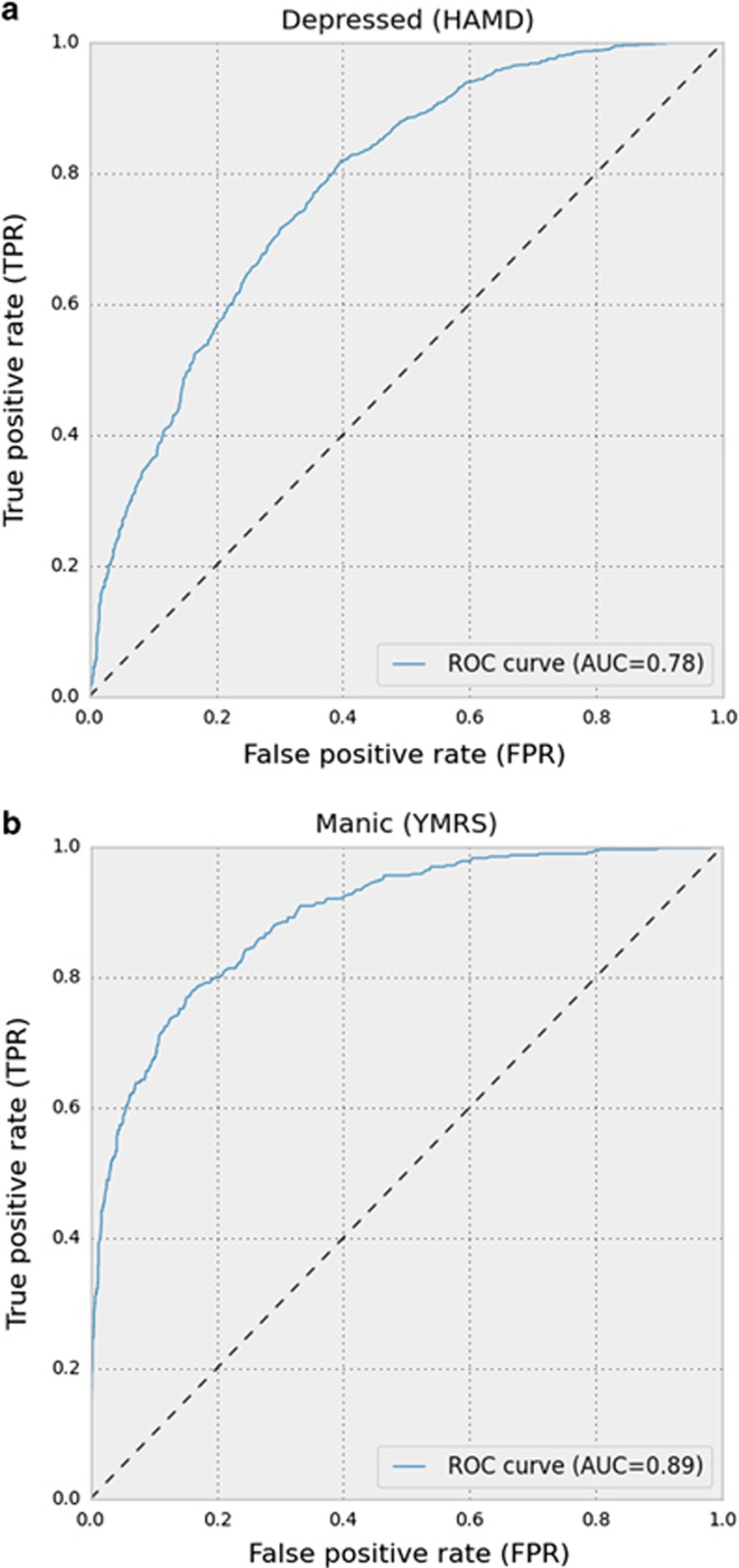
(**a**) Receiver operating curve (ROC) curve and area under the curve (AUC) based on user-independent models on voice data for classification of a depressive state versus a euthymic state. A depressive state was defined as a Hamilton Depression Rating Scale 17-item (HAMD) score ⩾13 and a Young Mania Rating Scale (YMRS) score <13. A euthymic state was defined as a HAMD<13 and an YMRS<13. (**b**) Receiver operating curve (ROC) curve and area under the curve (AUC) based on user-independent models on voice data for classification of a manic/mixed state versus a euthymic state. A manic or mixed state was defined as a Young Mania Rating Scale (YMRS) score ⩾13. A euthymic state was defined as a Hamilton Depression Rating Scale (HAMD) score <13 and an YMRS<13.

Table 3 presents the results for classification of affective states using voice features in user-dependent models, as well as user-independent models. The mean accuracy for classification of a depressive state versus a euthymic state based exclusively on voice data was 0.70 (s.d. 0.13) with a sensitivity of 0.64 (s.d. 0.25), and for a manic or mixed state versus a euthymic state the accuracy was 0.61 (s.d. 0.04) with a sensitivity of 0.71 (s.d. 0.09). Table 3 also presents the results of accuracy for classification of affective states using voice data in user-independent models. The accuracy for classification of a depressive state versus a euthymic state based exclusively on voice data was 0.68 (s.d. 0.006) with a sensitivity of 0.81 (s.d. 0.008), and for a manic or mixed state versus a euthymic state the accuracy was 0.74 (s.d. 0.005) with a sensitivity of 0.97 (s.d. 0.002). Table 3 also presents the specificity for all models. The corresponding ROC curves including AUC on classifications of a depressive and a manic or mixed state based on the user-independent models are presented in Figures 1a and b. The models classifying a depressive state versus a euthymic state had an AUC of 0.78 and models classifying a manic or mixed state versus a euthymic state had an AUC of 0.89.

### Combined voice features and automatically generated objective data for classification of affective states

Table 4A presents the results for classification of affective states using a combination of voice features and automatically generated objective data in user-dependent models, as well as user-independent models. The data set combining voice features and automatically generated objective data is different in size from the original data set on classification models using voice features exclusively, since automatically generated objective data were not always available for each data point in the voice data set. The results from models trained on voice features alone for every given data set are therefore also presented.

As can be seen from Table 4A, the accuracy, sensitivity and specificity were not increased when combining voice features with automatically generated objective data compared with exclusively using voice features.

### Combined voice features and daily electronic self-monitored data for classification of affective states

Table 4B presents the results for classification of affective states using a combination of voice features and daily electronic self-monitored data in user-dependent models, as well as user-independent models. As with the data presented in Table 4A, the data set combining voice features and daily electronic self-monitored data is different in size from the original data set on classification models using voice features exclusively, since electronic self-monitored data were not always available for each data point in the voice data set. The results from models trained on voice features alone for every given data set are therefore also presented.

As can been seen from Table 4B in the user-independent models, combining voice features and daily self-monitored data increased the accuracy, sensitivity and specificity compared with exclusively using voice features (see column in Table 4B).

### Combined voice features; automatically generated objective data; and daily electronic self-monitored data for classification of affective states

Table 4C presents the results for classification of affective states using a combination of all features, that is, voice features, automatically generated objective data and daily electronic self-monitored data in user-dependent models, as well as user-independent models. As with the data presented in Tables 4A and B, the data set combining voice features automatically generated objective data and daily electronic self-monitored data is different in size from the original data set on classification models exclusively using voice features, since automatically generated objective data and electronic self-monitored data were not always available for each data point in the voice data set. The results from models trained on voice features alone for every given data set are therefore also presented.

As can be seen from Table 4C, combining voice features, automatically generated objective data and self-monitored data increased the accuracy, sensitivity and specificity in three out of four analyses compared with exclusively using voice features. Comparing the combined data sets in Tables 4B and C, it can be seen that adding automatically generated objective data seems to give a small increase in accuracy, sensitivity and specificity compared with using and combination of voice features and daily self-monitored data.

## Discussion

In accordance with our hypotheses, we found that affective states in patients with bipolar disorder were classified by models based exclusively on voice features extracted during real-life phone calls in naturalistic settings. The analyses showed that voice features were more accurate in classifying manic or mixed states with an AUC=0.89 compared with an AUC=0.78 for the classification of depressive states.

Further, combining voice features and electronic self-monitored data increased the accuracy, sensitivity and specificity of classifying affective states slightly (Table 4B). Combining data on voice features and electronic self-monitored data with automatically generated objective data in the analyses also increased the accuracy, sensitivity and specificity of classifying affective states (Table 4B compared with Table 4C). Findings from the present study suggests that collecting data on alterations in speech accurately and with a high sensitivity can classify manic or mixed states in bipolar disorder, but less accurately classify depressive states. From the present study, it is not clear whether user-dependent models are superior to user-independent models in classifying affective states. Studies including more patients are necessary to clarify this issue.

The human voice is composed of multiple different components created through complex muscle movements making it individual for each person like ‘a fingerprint’. Interestingly, data from this innovative study shows that changes in voice features can in fact detect individual changes in affective state.

Strengths of the present study are that (1) a larger sample of patients (*N*=28) with bipolar disorder compared with previous studies was included,^17^, ^18^ (2) the study investigated the classification of affective states using a combination of voice features; automatically generated objective data on behavioral activities and electronic self-monitored data collected in real-time and naturalistic settings, (3) the study included patients presenting with depressive, as well as manic symptoms during follow-up, and (4) the affective states were classified using total scores on face-to-face golden standard clinicians administrated rating scales done by a researcher blinded to smartphone data.

The findings from the present study are in line with results from other studies. Karam *et al.*^17^ reported that (hypo)manic states (AUC: 0.81 (s.d. 0.17)) more accurately were classified than depressive states (AUC: 0.67 (s.d. 0.18)) using changes in voice features such as pitch. However, the included patients did not present with manic states during the follow-up period, the clinical assessments of affective states were phone-based (that is, the clinicians did not evaluate the patients face-to-face), and other electronic data such as automatically generated objective data and electronic self-monitored data were not collected.^17^ A study by Muaremi *et al.*^18^ reported that combining voice features (pitch) and automatically generated objective data (the number and duration of phone calls) in individual statistical models classified affective states with a mean accuracy of 0.82 (s.d. not reported). The study did not state how affective states were assessed and classified, it was not stated whether patients presented with depressive or manic/mixed states during follow-up, and the classification accuracy was not reported separately for depressive and manic states.

In longitudinal monitoring of affective symptoms in bipolar disorder, accurate classification of affective states based exclusively on voice features has great potential. The patients would not be required to fill out electronic self-monitoring on a daily basis but could still benefit from such a monitoring system by having the software installed on their smartphone. In addition, clinicians would get accurate and objective real-time data on the patients’ affective states based on collected voice features. This could provide opportunities for monitoring symptoms during long-term outside clinical settings and give possibilities for an individual intervention strategy between outpatient visits.

It has been estimated that one-third of the world’s population will use a smartphone by the year of 2017.^35^ Many people carry their phone with them during large parts of the day making it an essential part of their life, and many feel uncomfortable without their smartphone.^36^, ^37^ Thus, smartphones could represent a readily available, obvious, ideal and unobtrusive method for collecting continuous long-term data on illness activity in patients with bipolar disorder.

### Limitations

The study included a small sample of patients, but due to the design of the study with repeated measurements of each patient and collection of large amounts of smartphone data the statistical power was increased. Further, the follow-up period of the study could have been longer, allowing the patients to present with more affective episodes and more severe depressive and manic symptoms. However, the patients were included at the beginning of their course of treatment at the Copenhagen Clinic for Affective disorders, and the included patients presented with moderate to severe levels of depressive and manic symptoms during the follow-up period allowing for collection of data during different affective states.

During the recruitment phase, five patients declined to participate since the smartphone system was not available for iPhones. Patients using other smartphones than Android may represent a clinically different sub-group of patients than the one investigated in the present study. If possible, future studies should consider also supporting both iPhones and Windows smartphones, thereby enabling data collection from different types of smartphone operating systems. Also, future studies employing data analyses broken down by operating system and/or phone type to investigate a potential impact of the specific sensors used by iPhones as compared with Windows smartphones and/or Android smartphones on the accuracy and reliability of the data being collected would be interesting.

The patients were instructed to use their smartphones for usual communicative purposes during the study period and to carry the smartphones with them during the day. However, it cannot be excluded that some patients did not carry the smartphones with them at all times, calling from other devices and thereby not providing voice features during all their phone calls. However, the advantages of using smartphones for this kind of voice feature collection with low levels of intrusiveness and not a separate monitoring device seem to outweigh any potential missing data.

In the present study, patients’ affective states were defined according to an ICD-10 diagnosis of bipolar disorder current episode depressive, manic or mixed combined with the total score of depressive and manic symptoms ⩾13 according to the HAMD and the YMRS, respectively. We chose a cut-off on the HAMD and the YMRS of 13, respectively, to achieve a high validity of a current affective depressive or manic/mixed state. Consequently, a current euthymic state was defined as a HAMD and an YMRS <13 and in this way also including states with partial remission. In seven cases, the manic states also included depressive symptoms with a HAMD ⩾13, that is, a mixed state. Conversely, during depressive states the level of manic symptoms was low.

The large number of voice features collected in the present study proved to be a challenge in the statistical analyses. Other standard configurations than the openSMILE emolarge feature set are available, producing smaller sets of features.^15^ It would be relevant to compare the performance of other configurations of the openSMILE toolkit to the one used in the present study to investigate whether it could be feasible to reduce the feature set while keeping or improving the classification. This would help to reduce computational costs and save storage space.

From the present employed statistical analyses, it was not possible to extract which of the included automatically generated objective data that were the most contributing and useful. However, we have previously compared such correlations.^27^, ^28^

### Perspectives and future implications

To the best of our knowledge, this is the first study to investigate combinations of voice features; automatically generated objective data and electronic self-monitored data as state markers in patients with bipolar disorder. Using feature analysis collected in real-time from smartphones for classifying affective states in bipolar disorder reflects an innovative, objective and unobtrusive method for monitoring of illness activity (state) during long-term and in naturalistic settings.

Mobile health (mHealth) uses portable and wireless devices in the delivery of mental health services, and aims to improve access to services and improve quality of care. mHealth services are foreseen to have significant impact on mental healthcare to sense, analyze and modify human behavior.^38^, ^39^, ^40^ Big data analysis on voice features and automatically generated objective data that otherwise would be difficult to detect and measure could be collected using smartphones.^41^ Big data represent large amounts of data that are generated fast, have great variety and are complex.^42^ Furthermore, big data provides opportunities for exploration, observation and hypothesis generation, and analyses may lead to detection of new markers of illness activity in bipolar disorder.^43^, ^44^ Using smartphones to collect large amounts of data on personal behavioral aspects leads to possible issues on privacy, security, storage of data, safety, legal and cultural differences between nations that all should be considered, addressed and reported accordingly.^38^, ^40^, ^45^, ^46^, ^47^, ^48^, ^49^ Furthermore, employing statistical analyses on large data sets including large numbers of variables introduces an increased risk of false findings, and some of the explanatory variables may not be independent.^41^, ^45^, ^50^ Also, time varying confounding and exposure could be an issue, and future analyses should address and consider these issues.

## Conclusions

In patients with bipolar disorder, affective states were classified by sampling and analyzing voice features collected from smartphones used in real-time and naturalistic settings. The accuracy of classification of affective states based on voice features was in the range of 0.61–0.74, relying on both user-dependent and user-independent models. Combining voice features with automatically generated objective smartphone data on behavioral activities and electronic self-monitored data on illness activity increased the accuracy slightly. These results show that real-time collection and analysis of voice features from everyday phone calls may represent state markers in bipolar disorder and seem promising as a tool for continuous monitoring of illness activity and effect of treatment in patients with bipolar disorder.

## Figures and Tables

**Table 1:** Background and clinical characteristics of patients with bipolar disorder the MONARCA system for smartphones, *N*=28^a^

Age (years)	30.3 (9.3)
Female sex, % (*n*)	65.4 (18)
HAMD at inclusion	12.8 (5.4)
YMRS at inclusion	4.8 (5.3)
Number of depressive episodes	4 [2–6]
Number of (hypo)manic episodes	2 [1–5]
Number of hospitalizations	1 [0–2]
Bipolar disorder type I, % (*n*)	53.9 (16)
Age at onset (years)	20.9 (7.1)
Illness duration (years)	9.6 (6.3)
Years of education after primary school	4.8 (3.3)
	
*Social status*	
Employed full time,% (*n*)	8.0 (2)
Student, % (*n*)	40.0 (11)
Unemployed, % (*n*)	32.0 (8)
In relationship, % (*n*)	53.9 (15)
	
*Psychopharmacological treatment*	
Anticonvulsants, % (*n*)	46.2 (13)
Lithium, % (*n*)	46.2 (13)
Antipsychotics, % (*n*)	76.9 (22)
Antidepressants, % (*n*)	7.7 (2)

Abbreviations: HAMD, Hamilton Depression Rating Scale 17-item; IQR, inter-quartile range; YMRS, Young Mania Rating Scale.

a Data are mean (s.d.), median (IQR) or proportions (%, (*n*)) unless otherwise stated.

**Table 2:** Clinical assessments of the severity of depressive and manic symptoms according to standardized rating scales during different affective states in patients with bipolar disorder, *N*=179^a^

	*Depressive state,* n*=43*^b^	*Manic or mixed state,* n*=21*^c^	*Euthymic state,* n*=103*^d^
HAMD	17.1 (3.7)	11.2 (4.0)	7.0 (3.6)
YMRS	3.7 (3.1)	16.9 (3.4)	2.7 (3.0)

Abbreviations: HAMD, Hamilton Depression Rating Scale 17-item; YMRS, Young Mania Rating Scale.

a *N* represents the total number of clinical assessments with repeated measurements per patient during follow-up. Data are mean (s.d.) and unadjusted values.

b Defined as HAMD⩾13 and YMRS< 13.

c Defined as YMRS⩾13.

d Defined as HAMD<13 and YMRS<13.

**Table 3:** Classification of affective states based on voice features

	*Accuracy (s.d.)*^a^	*Sensitivity (s.d.)*^b^	*Specificity (s.d.)*^c^
*User-dependent models*^d^			
A depressive state^e^ versus a euthymic state^f^ (*n*=13)	0.70 (0.13)	0.64 (0.25)	0.75 (0.23)
A manic or mixed state^g^ versus a euthymic state^f^ (*n*=3)	0.61 (0.04)	0.71 (0.09)	0.50 (0.08)
			
*User-independent models*^d^			
A depressive state^e^ versus a euthymic state^f^	0.68 (0.006)	0.81 (0.008)	0.56 (0.008)
A manic or mixed state^g^ versus a euthymic state^f^	0.74 (0.005)	0.97 (0.002)	0.52 (0.01)

Abbreviations: HAMD, Hamilton Depression Rating Scale 17-item; YMRS, Young Mania Rating Scale.

Data are mean and s.d.

a Defined as accuracy= (true positive+true negative)/ (positive+negative).

b Defined as sensitivity= true positive/positive.

c Defined as specificity= true negative/negative.

d User-dependent models: building a model from each individual patient. User-independent models: building a common model from all patients.

e Defined as a HAMD score ⩾13 and a YMRS score <13.

f Defined as HAMD<13 and YMRS<13.

g Defined as a YMRS score ⩾13.

**Table 4:** Classification models of affective states based on combined smartphone data

	*Accuracy (s.d.)*^a^	*Accuracy (s.d.)*^a^	*Sensitivity (s.d.)*^b^	*Specificity (s.d.)*^c^
*A. Classification models of affective states based on a combination of voice features and automatically generated objective data*				
*User-dependent models*^d^				
A depressive state^e^ versus a euthymic state^f^ (*n*=6)	0.59 (0.09)	0.59 (0.10)	0.46 (0.21)	0.71 (0.13)
A manic or mixed state^g^ versus a euthymic state^f^ (*n*=3)	0.58 (0.03)	0.59 (0.02)	0.66 (0.11)	0.52 (0.13)
				
*User-independent models*^d^				
A depressive state^e^ versus a euthymic state^f^	0.62 (0.01)	0.62 (0.01)	0.78 (0.01)	0.47 (0.02)
A manic or mixed state^g^ versus a euthymic state^f^	0.72 (0.006)	0.73 (0.008)	0.95 (0.004)	0.51 (0.02)
				
*B. Classification models of affective states based on a combination of voice features and daily electronic self-monitored data*				
*User-dependent models*^d^				
A depressive state^e^ versus a euthymic state^f^ (*n*=9)	0.58 (0.12)	0.58 (0.13)	0.45 (0.21)	0.72 (0.19)
A manic or mixed state^g^ versus a euthymic state^f^ (*n*=3)	0.55 (0.03)	0.55 (0.02)	0.66 (0.11)	0.44 (0.16)
				
*User-independent models*^d^				
A depressive state^e^ versus a euthymic state^f^	0.62 (0.01)	0.66 (0.01)	0.77 (0.008)	0.55 (0.02)
A manic or mixed state^g^ versus a euthymic state^f^	0.72 (0.009)	0.75 (0.01)	0.96 (0.002)	0.53 (0.02)
				
*C. Classification models of affective states based on a combination of voice features, automatically generated objective data and daily electronic self-monitored data*				
*User-dependent models*^d^				
A depressive state^e^ versus a euthymic state^f^ (*n*=5)	0.60 (0.09)	0.62 (0.10)	0.45 (0.22)	0.78 (0.10)
A manic or mixed state^g^ versus a euthymic state^f^ (*n*=2)	0.55 (0.02)	0.58 (0.006)	0.71 (0.10)	0.45 (0.11)
				
*User-independent models*^d^				
A depressive state^e^ versus a euthymic state^f^	0.63 (0.009)	0.66 (0.01)	0.77 (0.01)	0.55 (0.02)
A manic or mixed state^g^ versus a euthymic state^f^	0.73 (0.01)	0.77 (0.01)	0.96 (0.005)	0.58 (0.03)

Abbreviations: HAMD, Hamilton Depression Rating Scale 17-item; YMRS, Young Mania Rating Scale.

Data are represented as mean and s.d.

a Defined as accuracy=(true positive+true negative)/(positive+negative).

b Defined as sensitivity=true positive/positive.

c Defined as specificity=true negative/negative.

d User-dependent models: building a model from each individual patient. User-independent models: building a common model from all patients.

e Defined as a HAMD score ⩾13 and a YMRS score <13.

f Defined as HAMD<13 and YMRS<13.

g Defined as a YMRS score ⩾13.
